# Effect of dietary magnolia bark extract supplementation in finishing pigs on the oxidative stability of meat

**DOI:** 10.1186/s40104-022-00740-0

**Published:** 2022-08-08

**Authors:** Ruggero Menci, Hajer Khelil-Arfa, Alexandra Blanchard, Luisa Biondi, Marco Bella, Alessandro Priolo, Giuseppe Luciano, Antonio Natalello

**Affiliations:** 1 Dipartimento Di Agricoltura, Alimentazione e Ambiente (Di3A), University of Catania, Via Valdisavoia 5, 95123 Catania, Italy; 2 Pancosma ADM, A One Business Center, 1180 Rolle, Switzerland

**Keywords:** Antioxidant capacity, Antioxidant enzyme, Color, Fatty acid, Lipid oxidation, Plant extract, Pork, Shelf-life, TBARS, Vitamin

## Abstract

**Background:**

Magnolia bark extract (MBE) is a natural supplement with antioxidant, anti-inflammatory, and antimicrobial activities. Its properties suggest that the dietary supplementation in livestock could improve the quality of products. Therefore, the aim of this study was to investigate, for the first time, the effect of dietary MBE supplementation (0.33 mg/kg) in finishing pigs on the oxidative stability of meat. Oxidative stability is of paramount importance for pork, as it affects storage, retail, and consumer acceptance. For the purpose, the fatty acid profile, cholesterol, fat-soluble vitamins, antioxidant enzymes (catalase, glutathione peroxidase, and superoxide dismutase), non-enzymatic antioxidant capacity (TEAC, FRAP, and Folin-Ciocalteu assays), color stability, and lipid stability of pork were assessed.

**Results:**

Concerning carcass characteristics, dietary MBE did not affect cold carcass yield, but reduced (*P* = 0.040) the chilling weight loss. The meat from pigs fed MBE had a lower (*P* = 0.031) lightness index than the control meat. No effect on intramuscular fat, cholesterol, and fatty acid profile was observed. Dietary MBE did not affect the content of vitamin E (α-tocopherol and γ-tocopherol) in pork, whereas it reduced (*P* = 0.021) the retinol content. The catalase activity was 18% higher (*P* = 0.008) in the meat from pigs fed MBE compared with the control group. The MBE supplementation reduced (*P* = 0.039) by 30% the thiobarbituric acid reactive substances (TBARS) in raw pork over 6 d of aerobic refrigerated storage. Instead, no effect on lipid oxidation was observed in cooked pork. Last, the meat from pigs fed MBE reduced Fe^3+^-ascorbate catalyzed lipid oxidation in muscle homogenates, with a lower (*P* = 0.034) TBARS value than the control group after 60 min of incubation.

**Conclusions:**

Dietary MBE supplementation in finishing pigs delayed the lipid oxidation in raw meat. This effect was combined with an increased catalase concentration. These results suggest that dietary MBE could have implications for improving the shelf-life of pork.

## Background

Nowadays, consumers’ choice is more and more driven by the natural and ecological implications of food production [1], even for pork. Swine farming system could indeed be used to position meat products on the market [2]. In this scenario, feeding pigs with plant extracts in place of artificial supplements fits the philosophy of a natural product. In addition, natural additives such as plant extracts (e.g., *Castanea sativa* Mill. extract) are allowed in organic farming [3].

Magnolia (*Magnolia officinalis*) bark extract (MBE) has been used in traditional Chinese, Japanese, and Korean medicine for thousands of years. Magnolia bark extract is acknowledged for several pharmacological activities, including benefits for the digestive, respiratory, cardiovascular, and central nervous systems [4]. The main bioactive compounds of MBE are magnolol and honokiol, two neolignans with antioxidant, anti-inflammatory, and antimicrobial activities [5, 6]. Furthermore, MBE is safe for dietary consumption and does not show any toxic or mutagenic potential in animal [7, 8]. Thanks to these properties, MBE has the potential of a natural feed additive with positive implications for animal health and product quality. For example, dietary MBE increased growth performance and transcripts for antioxidant enzymes in chickens [9]. Also, MBE supplementation is reported to modify the levels of certain metabolites absorbed in the chicken intestine [10].

Concerning swine and pork, although literature counts several studies about dietary plant extracts, such as *Saururus chinensis* (Lour.) Baill. [11], *Lippia* spp. [12], *C. sativa* [13], and *Saccharum officinarum* L. [14] extracts, the supplementation of MBE in pigs has never been investigated before. Our hypothesis was that magnolol, honokiol, and the others bioactive compounds present in MBE would enhance the oxidative stability of pork. Therefore, the aim of the present work was to analyze the effect of dietary MBE supplementation in finishing pigs on the fatty acid (FA) profile, fat-soluble vitamins, antioxidant enzymes, non-enzymatic antioxidant capacity, color stability, and lipid stability of meat, as well as growth and carcass performances.

## Methods

### Experimental design, animals, and diets

Castrated male pigs (crossbred PIC × Piétrain) were selected in a local commercial farm for their age (125 ± 5 d) and bodyweight (60.8 ± 3.80 kg). Twenty animals were transported to the facilities of the University of Catania and allocated in individual pens. All the pens were in the same room on a concrete floor with wheat straw bedding, and they were fitted with metal trough and nipple water dispenser. The experimental pigs were randomly assigned to two experimental groups (*n* = 10) and fed ad libitum a pelleted basal diet (Table 1) for 56 d. The diet of one of the groups was supplemented with 0.33 mg/kg of MBE (Pancosma S.A., Rolle, Switzerland) throughout the feeding trial. Before pelleting, MBE was pre-mixed with 1:1 (w:w) sepiolite and calcium carbonate and incorporated into the basal diet in the ratio of 1 kg/1000 kg, in order to ensure homogeneous distribution. The sepiolite-calcium carbonate carrier was also included in the control diet at the same dosage. Pigs were allowed free access to feed and water. To assess the feed consumption, offered feed and orts were recorded for each pig. Individual bodyweight was recorded every two weeks during the trial. Animals were handled by specialized personnel.

**Table 1:** Ingredients and chemical composition of the basal diet

Items	Content
Ingredients, g/kg as fed	
Maize	420
Barley	200
Wheat bran	152
Soybean meal (48% crude protein)	134
Fava bean	74
Vitamin-mineral premix^a^	10
Sodium carbonate	8
Amino acid premix^b^	2
Chemical composition^c^, g/kg DM	
DM, g/kg as fed	905
Crude protein	170
Crude fat	32.9
Neutral detergent fiber	165
Ash	41.7
Fatty acids^c^, g/kg DM	
C16:0	2.52
C18:0	0.49
C18:1 *c*9	3.50
C18:2 *c*9*c*12	7.61
C18:3 *c*9*c*12*c*15	0.58
Tocopherols^c^, mg/kg DM	
α-Tocopherol	7.57
γ-Tocopherol	21.5
δ-Tocopherol	6.40

^a^One kg of premix contained: vitamin A (650,000 U), vitamin D_3_ (200,000 U), vitamin E (7000 mg), vitamin K_3_ (250 mg), vitamin B_1_ (250 mg), vitamin B_2_ (450 mg), vitamin B_6_ (350 mg), vitamin B_12_ (3 mg), niacinamide (2500 mg), calcium D-pantothenate (2000 mg), folic acid (100 mg), choline chloride (50,000 mg), iron(II) sulfate monohydrate (10,000 mg); manganous sulphate monohydrate (7500 mg); copper(II) sulphate pentahydrate (1500 mg); potassium iodide (100 mg); sodium selenite (30 mg)

^b^One kg of premix contained: lysine (80,000 mg), threonine (280,000 mg), methionine (240,000 mg), tryptophan (120,000 mg), L-valine (240,000 mg)

^c^Analyzed as described in the Methods section

### Slaughtering and sampling

At the end of the feeding trial (i.e., after 56 d), all the pigs were slaughtered on the same day by electric stunning and exsanguination, according to the European Union Regulation (council regulation EC No. 1099/2009). Slaughtering took place in a near commercial abattoir, about 30 min away from the experimental stable. Hot carcass weight was measured within 20 min from slaughter. Muscle pH was measured in the *longissimus thoracis et lumborum* muscle (LTL) after 45 min post-mortem, using a pH-meter outfitted with a penetrating electrode (Orion 9106; Orion Research Incorporated, Boston, MA). Carcasses were stored for 24 h at 0–4 °C, then cold carcass weight and muscle pH (as above) were assessed.

A portion of LTL muscle (between the 13^th^ thoracic vertebra and the 3^rd^ lumbar vertebra), together with backfat and rind stuck on it, was excised from the right side of each carcass. Samples were vacuum packaged and transported refrigerated to the laboratories of the University of Catania, where all the analyses were performed. On the same day, the color parameters of backfat and meat were measured by a portable spectrophotometer (CM-2022, Minolta Co. Ltd. Osaka, Japan) set to operate in SCE mode (illuminant A, 10° standard observer). Three measurements were taken on the surface on non-overlapping areas and averaged. The color descriptors L* (lightness), a* (redness) and b* (yellowness) were measured in the CIE L*a*b* color space. The samples of LTL were then trimmed from backfat and cut into three aliquots: i) a first aliquot, intended for FA determination, was vacuum-packed and stored at −80 °C; ii) a second aliquot was chopped and immediately frozen in liquid nitrogen and stored at −80 °C, pending analyses of cholesterol, fat-soluble vitamins, antioxidant enzymes, and non-enzymatic antioxidant capacity; iii) a third aliquot, intended for oxidative stability assessment, was vacuum-packed and aged for 24 h at 0–4 °C.

Sub-samples of the basal diet were collected three times over the experiment, mixed thoroughly, and stored vacuum-packaged at −20 °C, pending analyses.

### Feed analyses

After grounding in a hammer mill (1-mm screen), a basal diet sample was analyzed for dry matter (DM), crude protein, crude fat, and ash, according to the AOAC [15] methods, and neutral detergent fiber, according to Van Soest et al. [16].

Fatty acids profile of the basal diet was assessed following the method of Valenti et al. [17], through a one-step extraction-transesterification. Briefly, 100 mg of ground sample was placed into a tube, and 1.5 mL of chloroform and 2.5 mL of methanolic sulfuric acid (2%) in methanol were added. The tube was incubated in a water bath at 70 °C for 2 h and cooled to room temperature. Then, 1.5 mL of chloroform and 2.5 mL of 6% K_2_CO_3_ were added, and the tube was centrifuged at 2500 × *g* for 10 min at 4 °C. One mL of the underlying organic phase was collected, evaporated under nitrogen flow, and then dissolved in 1 mL of hexane. Gas-chromatographic analysis was performed as later described for intramuscular FA. Tridecanoic acid (C13:0) was used as internal standard.

Tocopherols of the basal diet were extracted from a 200-mg ground sample using 3 mL of methanol:acetone:petroleum ether (1:1:1, v:v:v) with BHT (0.1 g/L), and vortexing 1 min [18]. After centrifugation at 1000 × *g* for 5 min, the supernatant was collected. The extraction procedure was performed 3 times. Then, the supernatant was evaporated under nitrogen flow and the residue was dissolved in 1 mL of methanol. The residue was filtered with a 0.22-µm PTFE filter and placed into a 2-mL vial. Tocopherols were determined through ultra-high-performance liquid chromatograph (UHPLC) as later described for meat.

### Meat analyses

#### Intramuscular fat and fatty acid profile

Intramuscular fat was extracted from 10-g meat samples using 2:1 (v:v) chloroform:methanol [19]. Then, FA were converted to fatty acid methyl esters through basic transesterification using sodium methoxide in methanol. The FA profile of meat was then assessed using a gas-chromatograph (TRACE GC; Thermo Finnigan, Milan, Italy) equipped with a high-polar fused silica capillary column (length: 100 m; i.d.: 25 mm; film thickness: 0.25 μm; SP. 24,056; Supelco Inc., Bellefonte, PA) and a flame ionization detector. Instrument setting was the same described by Natalello et al. [20]. Methyl nonadecanoate (C19:0) was used as internal standard.

#### Cholesterol and fat-soluble vitamins

Cholesterol, tocopherols, and retinol in meat were determined using the method developed by Bertolín et al. [21], with minor changes. After freeze-drying, 500-mg meat samples were placed into centrifuge tubes. Then, 200 mg of L-ascorbic acid and 7.5 mL of 10% KOH in ethanol:water (1:1, v:v) were added. Saponification took place overnight at 22 °C in an incubator shaker (KS 4000 i control, IKA®-Werke GmbH & Co. KG, Staufen, Germany) set at 250 r/min and protected from light. The following day, 5 mL of hexane:ethyl acetate (9:1, v:v) with BHT (25 mg/L) was added, the tubes were vortexed for 60 s and centrifuged at 2000 × *g* for 5 min at 10 °C (Centrifuge 5810 R; Eppendorf s.r.l., Milano, Italy), and supernatants were collected. This operation was performed twice. The supernatants were evaporated under nitrogen flow at 40 °C using a sample concentrator and a block heater (SBHCONC/1 and SBH130D/3 Stuart®; Cole-Parmer, Stone, United Kingdom). Dry residues were dissolved in 1 mL of methanol (HPLC grade), warming tubes at 40 °C and vortexing thoroughly, and then filtered with 0.2-µm/13-mm PTFE syringe filters and placed into 2-mL amber vials. For identifying and quantifying the analytes, 10 µL of extract was injected in a Nexera UHPLC (Shimadzu Corporation, Kyoto, Japan) featuring a C18 phase column (Zorbax ODS; length: 25 cm; i.d.: 4.6 mm; particle size: 5 µm; Supelco, Bellefonte, PA), with methanol as isocratic mobile phase (flow rate: 1.3 mL/min). Samples were kept at a temperature of 25 °C (SIL-40C XS Autosampler, Shimadzu) and the column oven was set at 40 °C (CTO-40C, Shimadzu). Cholesterol and retinol were detected with a photodiode array detector (SPD-M40, Shimadzu) at the absorbance wavelength of 220 nm and 325 nm, respectively. Tocopherols were detected by fluorescence (RF-20AXS, Shimadzu) with excitation and emission wavelengths of 295 nm and 330 nm, respectively. The analytes were identified by comparison of retention times with those of pure standards (Merck Life Science s.r.l., Milano, Italy), and quantified with external calibration curves made with pure standards.

#### Antioxidant enzymes

The activities of catalase (CAT), glutathione peroxidase (GPx), and superoxide dismutase (SOD) were determined as described by Natalello et al. [22]. Each analysis was performed on a meat extract thus obtained. First, 5 g of frozen meat samples in 10 mL of ice-cold 50 mmol/L phosphate buffer (pH 7.0) was homogenized at 13,000 r/min for 60 s using a T18 digital Ultra-Turrax® (IKA®-Werke GmbH & Co. KG, Staufen, Germany). The tubes containing samples were always kept in a water/ice bath. After centrifugation at 2800 × *g* for 20 min at 4 °C, supernatants (1.8 mL) were collected in microtubes and centrifuged at 10,000 × *g* for 10 min at 4 °C. Finally, the meat extract was aliquoted in 3 microtubes and immediately frozen at −80 °C until enzymatic analyses.

The CAT activity was determined following the method described by Jin et al. [23], with some modifications. Briefly, 30 µL of meat extract was placed in a UV cuvette and 1.74 mL of H_2_O_2_ solution (11 mmol/L H_2_O_2_ in 50 mmol/L phosphate buffer) was added. The cuvette was immediately inverted 4 times, and the absorbance at 240 nm was monitored over 3 min in kinetics mode (model UV-1601; Shimadzu corporation, Kyoto, Japan) with reading interval of 1 s. The blank was prepared with 50 mmol/L phosphate buffer in place of meat extract. The CAT activity was calculated using the molar extinction coefficient of H_2_O_2_ (3.95 m^2^/mol). One unit (U) of CAT activity corresponds with the amount of enzyme needed to decompose 1 µmol of H_2_O_2_ per min.

The GPx activity was measured according to Flohé and Günzler [24], with minor adjustments. The meat extract was diluted 4 times in 50 mmol/L phosphate buffer, and 100 µL of this solution were mixed with 500 µL of assay medium (100 mmol/L pH 7.0 potassium phosphate buffer, 1 mmol/L EDTA, 2 mmol/L NaN_3_), 100 µL of glutathione reductase (2.4 U/mL), 100 µL of 10 mmol/L L-glutathione, and 100 µL of NADPH solution (1.5 mmol/L NADPH in 0.1% NaHCO_3_) in a UV cuvette. After 5 min of incubation at room temperature, 100 µL of 1.5 mmol/L H_2_O_2_ was added to start the reaction, and the absorbance at 340 nm was monitored for 5 min with reading interval of 1 s. A blank was prepared with 50 mmol/L phosphate buffer in place of meat extract. The GPx activity was calculated using the molar extinction coefficient of NADPH (622 m^2^/mol). One U of GPx activity corresponds with the amount of enzyme needed to oxidize 1 µmol of NADPH per min.

The SOD activity was evaluated following the method of Gatellier et al. [25]. In short, 20 µL of meat extract and 20 µL of 10 mmol/L pyrogallol were added into a UV cuvette containing 760 µL of 50 mmol/L Tris–HCl buffer (8.2 pH). After inverting the cuvette, the absorbance at 340 nm was monitored in kinetics mode for 300 s with reading interval of 1 s. A blank was prepared with 50 mmol/L phosphate buffer in place of meat extract. One U of SOD activity was defined as the amount of enzyme needed to inhibit the pyrogallol autoxidation by 50% through comparison with the blank.

The activity of each antioxidant enzyme in meat was expressed as U/g.

#### Non-enzymatic antioxidant capacity assays

To evaluate the radical scavenging activity and the reducing capacity of meat, the antioxidant capacity of meat aqueous extracts was estimated with three different non-enzymatic assays, as reported by Luciano et al. [26]. In brief, 1 g of minced meat in 10 mL of distilled water was homogenized at 9000 r/min for 1 min (Diax 900, Heidolph ElektroGmbH & Co. KG, Kelheim, Germany). The tubes containing samples were constantly kept in a water/ice bath during the homogenization. The samples were centrifuged at 2500 × *g* for 20 min at 4 °C, and supernatants were collected and filtered with Whatman paper (grade 541). The obtained meat extract was aliquoted into 3 subsamples, one for each of the following analyses, and stored at −80 °C.

Radical scavenging capacity was measured with the Trolox equivalent antioxidant capacity (TEAC) assay described by Aouadi et al. [27], with some modifications. Briefly, 20 µL of meat extract were mixed with 2 mL of ABTS [2,2-azinobis-(3-ethylbenzothiazoline 6-sulphonate)] radical solution and incubated in water bath at 30 °C for 60 min. Then, the absorbance at 734 nm of the samples was registered (UV-1601; Shimadzu corporation, Kyoto, Japan). The spontaneous discoloration of samples was corrected analyzing a blank prepared with 20 µL of distilled water in place of meat extract. A 4-points calibration curve (from 100 to 400 µg/mL) was made through analysis of a Trolox (238,813; Merck Life Science s.r.l., Milano, Italy) solution in phosphate buffer [pH 7.0; disodium phosphate dodecahydrate (Na_2_HPO_4_ × 12H_2_O) and KH_2_PO_4_].

Reducing capacity was measured with the ferric reducing antioxidant power (FRAP) assay developed by Benzie and Strain [28], with minor adaptations. In short, 50 µL of meat extract was mixed with 150 µL of distilled water and 1.5 mL of a solution 10:1:1 (v:v:v) of 300 mmol/L acetate buffer (pH 3.6), 10 mmol/L TPTZ solution (2,4,6-tripyridyl-s-triazine in 40 mmol/L HCl), and 20 mmol/L aqueous FeCl_3_. After incubation at 37 °C for 60 min, the absorbance of samples was read at 593 nm. A 6-points calibration curve was built with aqueous ferrous sulfate heptahydrate (from 28 to 280 µg/mL).

The reducing capacity of meat was also measured with the Folin-Ciocalteu assay described by Makkar et al. [29]. One mL of meat extract was diluted 1:2 with distilled water, and then mixed with 0.5 mL of 1 N Folin-Ciocalteu reagent and 2.5 mL of 20% sodium carbonate. The mixture was incubated in the dark for 40 min at room temperature and then centrifuged at 2500 × *g* for 10 min at 4 °C. The absorbance at 725 nm of the samples was registered. A 6-points calibration curve was made using aqueous tannic acid (from 10 to 100 µg/mL).

#### Oxidative stability

The oxidative stability of fresh and cooked meat was assessed over aerobic storage as reported by Valenti et al. [30]. From each sample of LTL, 3 slices were cut (thickness: 2 cm) and placed in polystyrene trays, which were then overwrapped with plastic wrap. The trays were stored in the dark at 0–4 °C. After 2 h (d 0), 3 d, and 6 d, respectively, one of the 3 slices was analyzed for L*, a*, b*, and the reflectance spectrum between 400 and 700 nm with a portable spectrophotometer (as described above in “Slaughtering and sampling”). The slice was then frozen pending lipid oxidation analysis. Similarly, 3 more slices of LTL were cut, vacuum packaged, and cooked in water bath at 70 °C for 30 min. The cooked meat was unpacked and weighted to measure the cooking weight loss. Then, one slice was immediately frozen pending lipid oxidation analysis, whereas the other two were first stored at 0–4 °C (as above) for 2 d and 4 d, respectively.

Afterward, the measurement of thiobarbituric acid reactive substances (TBARS) was used to assess the lipid oxidation in raw and cooked meat, following the procedure described by Natalello et al. [22]. In brief, 5-g samples were homogenized with 15 mL of 7.5% trichloroacetic acid and filtered using Whatman paper (grade 1). Four mL of the obtained liquid was reacted with 4 mL of 0.02 mol/L aqueous thiobarbituric acid and then incubated at 80 °C for 90 min. The absorbance at 532 nm was read, and the results were expressed as mg of malondialdehyde (MDA) per kg of meat, through comparison with a TEP (1,1,3,3-tetraethoxypropane) calibration curve (points ranging from 1.25 to 16.25 mmol/L).

The catalysis by Fe^3+^ and ascorbate (Fe-Asc) was used as further oxidative challenge for meat [26]. Ten g of raw frozen meat in 40 mL of MES [2-(N-morpholino)ethanesulfonic acid] buffer (pH 5.7) was homogenized and equilibrated at 0–4 °C. After collecting a 4-mL aliquot (time 0), 40 μL of an equimolar solution of ferric chloride hexahydrate and L‑sodium ascorbate was added (to a final concentration of 45 µmol/L), and samples were incubated at room temperature. After 30 and 60 min of incubation, other two 4-mL aliquots were collected. Each of the three aliquots was mixed with 4 mL of 15% trichloroacetic acid and filtered through Whatman paper (grade 1). The liquid obtained (2 mL) was reacted with 2 mL of 0.02 mol/L aqueous thiobarbituric acid and then used for TBARS determination, as above.

### Calculations and statistical analysis

Atherogenicity index and thrombogenicity index were calculated according to Ulbricht and Southgate [31]. The hypocholesterolemic to hypercholesterolemic ratio (h/H) was calculated as reported by Santos-Silva et al. [32]. Concerning color parameters, total color change (ΔE) after 3 and 6 d of storage was calculated as ΔE = [(ΔL*)^2^ + (Δa*)^2^ + (Δb*)^2^]^1/2^, where ΔL*, Δa*, and Δb* are the differences in L* (lightness), a* (redness), and b* (yellowness), respectively, between d 0 and d 3 or d 6. The ratio between the reflectance spectrum of raw meat at 630 nm and 580 nm (630/580) was calculated as indicator of myoglobin oxidation.

All the data were analyzed with the SPSS For Analytics (version 26; IBM corporation, Armonk, NY). The single animal was the statistical unit. The effect of dietary treatment on growth performance, carcass traits, intramuscular FA, fat-soluble vitamins, antioxidant enzymes, and non-enzymatic antioxidant capacity was statistically analyzed using one-way ANOVA. Differences were considered significant when *P* ≤ 0.050. Data of color and lipid stability were analyzed using a mixed model for repeated measures, with the single animal included as random factor. The fixed factors in the model were the dietary treatment, the time of storage/incubation, and their interaction. The Tukey post hoc test for multiple comparisons was performed when *P* ≤ 0.050.

## Results and discussion

### Growth performance, carcass traits, and meat physical properties

Table 2 displays data on growth performance, muscle pH, carcass weight and yield, backfat thickness, meat and backfat color, and meat cooking loss. Dietary MBE did not affect (*P* > 0.050) feed intake nor the average daily gain and feed conversion ratio of finishing pigs, which were consistent with Italian swine farming [33]. The carcasses of the MBE-fed pigs showed lower (*P* = 0.040) chilling weight loss compared with the control group. This effect is indeed due to the MBE supplementation, as slaughtering and carcass chilling took place on the same day at the same conditions. Moreover, backfat thickness, that could affect chilling rate and moisture loss, was similar (*P* > 0.050) between the two dietary treatments. Muscle pH too has a great influence on the ability of meat to retain water [34], but its effect can be ruled out as there was no difference (*P* > 0.050) between dietary treatments, and pH values after 45 min and 24 h were normal for pork [35]. Therefore, basing on our data, we cannot confidently explain the effect of dietary MBE on chilling weight loss of pig carcasses. Probably, dietary MBE may have regulated the intestinal absorption of metabolites that induce high water-holding capacity in muscle. Indeed, Park et al. [10] observed a significant variation in the concentration of over 250 metabolites, including amino acids, fatty acids, peptides, and nucleosides, in the intestine of chickens whose diet was supplemented with 0.33 mg/kg of MBE. In addition, Zou et al. [36] suggested that feeding antioxidants (such as MBE) – could reduce the oxidative damage to muscle cell membranes, thus preserving their ability to retain water. However, the significant difference in chilling weight loss did not lead to any difference (*P* > 0.050) in carcass weight and yield (both hot and cold) as well as in meat cooking loss between the two experimental groups.

**Table 2:** Effect of dietary magnolia bark extract on growth performance, carcass traits, and meat physical properties

Items	Dietary treatment		SEM	*P*-value
	CON	MBE		
Growth performance				
Average daily intake, kg/d	3.17	3.07	0.047	0.295
Final body weight, kg	115	113	1.16	0.275
ADG, kg/d	0.97	0.92	0.022	0.322
FCR	3.29	3.34	0.053	0.632
Carcass traits				
Muscle pH at 45 min	6.18	6.25	0.039	0.326
Muscle pH at 24 h	5.50	5.60	0.034	0.171
Hot carcass weight, kg	92.7	90.8	1.01	0.362
Carcass yield (hot), %	80.6	80.7	0.253	0.802
Cold carcass weight, kg	88.2	87.2	1.04	0.643
Carcass yield (cold), %	76.6	77.5	0.331	0.222
Chilling weight loss, %	4.85	3.99	0.214	0.040
Backfat thickness, cm	2.20	2.19	0.069	0.944
Meat color descriptors				
L* (lightness)	52.5	49.5	0.704	0.031
a* (redness)	7.73	7.69	0.217	0.931
b* (yellowness)	7.98	7.52	0.306	0.465
Backfat color descriptors				
L* (lightness)	75.1	73.3	0.541	0.105
a* (redness)	5.73	6.75	0.287	0.075
b* (yellowness)	6.35	7.70	0.372	0.069
Meat cooking loss, %	29.7	29.0	0.672	0.616

*ADG* Average daily gain, *CON* Basal diet (control group), *FCR* Feed conversion ratio (daily intake/ADG), *MBE* Basal diet supplemented with 0.33 mg/kg of magnolia bark extract, *SEM* Standard error of the mean

Concerning color, dietary MBE reduced (*P* = 0.031) meat lightness, whereas it did not affect (*P* > 0.050) the other color parameters of meat and backfat (Table 2). Studies reporting the effect of dietary plant extracts on pork quality did not show any changes in color parameters [12–14], except for Ao et al. [11], who instead observed a higher L* value after feeding pigs 2 g/kg of *S. chinensis* extract for 7 weeks. Consistent with our results, Zhang et al. [37] found a darker meat in pigs eating 300 or 600 mg/kg of resveratrol (extracted from *Reynoutria japonica* Houtt.). In their study, dietary resveratrol also enhanced the expression of fast oxido-glycolytic muscle fibers (type IIA), known to be darker. In any case, the L* value of meat does not contribute to variation in consumers’ choice as long as it falls within the normal values for pork [38], as in the present study.

### Intramuscular fat

Table 3 shows the intramuscular fat content, cholesterol content, and FA profile of pork: the values found in the present study are in line with literature [39, 40]. The supplementation of MBE in the diet of finishing pigs had no effect (*P* > 0.050) on these parameters. Accordingly, no study about dietary plant extracts observed an effect on the intramuscular fat content of pork [11, 41, 42]. Also, Rossi et al. [12] did not found any differences in the cholesterol content of meat from pigs receiving dietary *Lippia* spp. extract supplementation compared with the control group.

**Table 3:** Effect of dietary magnolia bark extract on pork intramuscular fat, cholesterol, and fatty acid profile

Items	Dietary treatment		SEM	*P*-value
	CON	MBE		
Intramuscular fat, g/100 g	1.65	1.62	0.103	0.896
Cholesterol, mg/g	0.60	0.52	0.022	0.055
Fatty acids, mg/100 g				
C10:0	2.22	1.87	0.158	0.285
C12:0	1.49	1.39	0.118	0.679
C14:0	20.2	19.3	1.46	0.770
C16:0	387	381	25.0	0.912
C17:0 *anteiso*	4.76	4.41	0.302	0.576
C16:1 *c*9	51.0	52.1	3.74	0.892
C17:0	2.35	2.53	0.169	0.602
C18:0	200	185	14.2	0.606
C18:1 *t*9	2.23	2.47	0.214	0.584
C18:1 *c*9	650	648	44.9	0.983
C18:1 *c*11	65.4	66.7	4.13	0.880
C18:2 *c*9*c*12	177	170	8.01	0.678
C20:0	2.69	2.40	0.284	0.621
C20:1 *c*11	11.0	11.5	0.822	0.756
C18:3 *c*9*c*12*c*15	6.35	5.36	0.512	0.345
C20:2 *c*11*c*14	5.10	5.73	0.458	0.506
C20:3 *n-*6	4.48	4.52	0.248	0.927
C20:3 *n-*3	0.80	0.67	0.148	0.677
C20:4 *n-*6	29.5	29.1	1.08	0.845
C22:4 *n-*6	4.37	4.60	0.193	0.562
C22:5 *n-*3	4.20	3.11	0.283	0.051
C22:6 *n-*3	0.86	1.04	0.248	0.727
Sums and calculations				
SFA	621	598	41.2	0.791
MUFA	779	781	53.3	0.992
PUFA	233	225	9.70	0.674
PUFA *n-*6	221	214	9.20	0.736
PUFA *n-*3	12.2	10.2	0.710	0.157
PUFA *n-*6/*n-*3	18.4	22.4	1.14	0.077
AI^a^	0.46	0.46	0.005	0.761
TI^b^	1.08	1.06	0.014	0.521
h/H^c^	2.38	2.38	0.024	0.943
HP-PUFA^d^	50.6	48.4	1.99	0.548
Peroxidability index^e^	328	316	15.2	0.617

*CON* Basal diet (control group), *MBE* Basal diet supplemented with 0.33 mg/kg of magnolia bark extract, *MUFA* Monounsaturated fatty acids, *PUFA* Polyunsaturated fatty acids, *SEM* Standard error of the mean, *SFA* Saturated fatty acids

^a^Atherogenicity index = (C12:0 + 4 × C14:0 + C16:0)/(MUFA + PUFA *n*-6 + PUFA *n*-3)

^b^Thrombogenicity index = (C14:0 + C16:0 + C18:0)/(0.5 × C18:1 + 0.5 × other MUFA + 0.5 × PUFA *n*-6 + 3 × PUFA *n*-3 + PUFA *n*-3/PUFA *n*-6)

^c^Hypocholesterolemic to hypercholesterolemic ratio = (sum of C18:1 *c*9, C18:1 *c*11, C18:2 *c*9c12, C20:1 *c*11, C18:3 *c*9*c*12*c*15, C20:2 *c*11*c*14, C20:3 *n-*6, C20:3 *n*-3, C20:4 *n*-6, C22:4 *n*-6, C22:5 *n*-3, C22:6 *n*-3)/(C14:0 + C16:0)

^d^Highly peroxidizable-PUFA, calculated as the sum of PUFA with three or more unsaturated bonds

^e^Peroxidability index = (Σ dienoic × 1 + Σ trienoic × 2 + Σ tetraenoic × 3 + Σ pentaenoic × 4 + Σ hexaenoic × 5)

The FA profile of pork reflects that of the diet, especially regarding polyunsaturated fatty acids [39]. Therefore, it is relatively easy to change the FA content of pork through the supplementation of different fat sources whereas non-fat supplements would hardly have effect. Indeed, studies investigating dietary plant extracts in pigs did not observe any variation in meat FA profile [11, 41, 42]. Mason et al. [43] reported a reduction in *n*-6 to *n*-3 ratio in the meat from pigs fed green tea catechins compared with the control group, but the two dietary treatments had very different fat sources: rapeseed oil and lard, respectively. Nonetheless, assessing the FA profile of meat is essential in oxidative stability studies, as the unsaturation level of fat is crucial to determine its proneness to oxidation. Particularly with dietary MBE, as it was recently demonstrated to affect the level of certain dietary FA in the chicken intestine, for example by increasing the presence of C17:0, C22:0, and C24:1 *n*-9 [10]. However, in the present study, pork FA profile did not differ between the two experimental groups and could thus be considered irrelevant to a possible effect of MBE on pork oxidative stability.

### Meat antioxidant capacity

The antioxidant capacity of meat depends on several cofactors working together to delay the oxidation process. The content of α-tocopherol, γ-tocopherol, retinol, the activity of antioxidant enzymes (i.e., CAT, GPx, and SOD) in pork as well as the non-enzymatic antioxidant capacity are reported in Table 4.

**Table 4:** Effect of dietary magnolia bark extract on the antioxidant capacity of pork

Items	Dietary treatment		SEM	*P*-value
	CON	MBE		
Fat-soluble vitamins				
α-tocopherol, µg/g of meat	3.16	3.17	0.117	0.961
γ-tocopherol, µg/g of meat	0.20	0.16	0.013	0.191
retinol, ng/g of meat	15.1	11.4	0.836	0.021
Antioxidant enzymes, U/g of meat				
Catalase (CAT)	141	166	4.93	0.008
Glutathione peroxidase (GPx)	0.26	0.27	0.012	0.723
Superoxide dismutase (SOD)	132	128	2.27	0.364
Non-enzymatic antioxidant capacity				
TEAC^a^	49.9	43.3	3.49	0.359
FRAP^b^	32.7	35.1	1.76	0.496
Folin–Ciocalteu^c^	0.69	0.65	0.023	0.317

*CON* Basal diet (control group), *MBE* Basal diet supplemented with 0.33 mg/kg of magnolia bark extract, *SEM* Standard error of the mean

^a^TEAC, Trolox equivalent antioxidant capacity. Expressed as mg of Trolox equivalents per g of meat

^b^FRAP, ferric reducing antioxidant power. Expressed as mg of Fe^2+^ equivalents per g of meat

^c^Expressed as mg of tannic acid equivalents per g of meat

Tocopherols are commonly considered as the main fat-soluble antioxidant in mammals, acting against lipid oxidation. Retinol is another fat-soluble vitamin with a number of biological activities and a certain antioxidant capacity, secondary to α-tocopherol [44]. The MBE supplementation in finishing pigs did not affect (*P* > 0.050) the tocopherols content of meat, whereas it reduced (*P* = 0.021) the retinol content (Table 4). A lack of effect of dietary plant extracts on α-tocopherol in pork was also observed by González and Tejeda [41] and Haak et al. [42]. To our knowledge, no information about the effect of any dietary plant extract on the retinol content in pork is available in literature. The metabolism of vitamin A is a complex biological process that involves hepatic storage and mobilization [45]. According to the findings of Olivares et al. [46], the content of retinol in pig *longissimus dorsi* muscle is independent from hepatic retinol when animals are fed practical levels of vitamin A (i.e., between 1300 and 13,000 U/kg of diet). Indeed, even if they observed a fivefold increase in hepatic retinol when feeding the highest dose of vitamin A, the retinol content of muscle did not change. Therefore, we could hypothesize that magnolol, honokiol, or some minor bioactive component of MBE may have affected the biological pathways for extrahepatic uptake of retinol, leading to a lower retinol content in muscle.

CAT, GPx, and SOD are the main enzymes involved in the cell defense from reactive oxygen species. The activities of CAT and GPx found in the present study were similar to those reported by Hernández et al. [47] in pig muscle, whereas the SOD activity was lower. However, the activity of these enzymes increases with age [48] and the pigs in the study of Hernández et al. [47] were slaughtered at 12 months of age, compared to the 6 months of the present study. We did not observe any difference (*P* > 0.050) in GPx and SOD activities between the two experimental groups, whereas the activity of CAT was higher (*P* = 0.008) in the MBE group (Table 4). This result on CAT activity can be explained in several ways. For instance, Rajgopal et al. [49] found MBE to activate the gene expression of *Nrf2* (nuclear factor E2-related factor 2) in cultured cells, which stimulates the enzymatic defense against hydrogen peroxide-mediated oxidative stress. The *Nrf2* activation was also confirmed by Lu et al. [50] studying pure magnolol. Moreover, Oh et al. [9] reported an increment in CAT genetic transcripts in chicken fed 0.33 or 0.56 mg/kg of MBE compared with the control group. Therefore, the increment in CAT activity observed in the present study was likely due to the dietary supplementation of MBE, probably due to its magnolol content.

The measurement of non-enzymatic antioxidant capacity by TEAC, FRAP, and Folin-Ciocalteu assays completes the picture of meat antioxidant capacity. Indeed, these assays added different information, excluding the antioxidant activities of enzymes and fat-soluble vitamins (as the analyses were performed on hydrophilic extract). In the present study, the MBE supplementation did not affect (*P* > 0.050) TEAC, FRAP, and Folin-Ciocalteu values (Table 4).

Comparison with literature is difficult, as only few studies investigating the effect of dietary plant extracts on pork quality assessed the non-enzymatic antioxidant capacity, and there is a wide diversity of experimental designs. For example, Zhang et al. [37] observed a higher antioxidant capacity in the meat of finishing pigs eating 300 or 600 mg of plant-origin resveratrol per kg of diet for 19 d, compared with the control group.

### Meat oxidative stability

Oxidative stability is of paramount importance for the quality of meat, as it involves storage, retail, and consumer acceptance. Table 5 shows the changes in raw pork color parameters during refrigerated storage and the development of lipid oxidation (as TBARS values) in raw, Fe-Asc catalyzed, and cooked pork. The MBE supplementation had no effect (*P* > 0.050) on pork color parameters, except for L*, which was confirmed to be lower (*P* = 0.004) in the MBE group than in the control group over 6 d of refrigerated storage. As expected, storage time affected (*P* < 0.001) the color parameters: L* was higher after 6 d than after 3 and 0 d of storage, whereas a*, b*, and 630/580 decreased from 0 to 3 d and from 3 to 6 d of storage. As a result, the overall color variation (ΔE) was higher (*P* = 0.002) after 6 d then after 3 d of storage. No interaction between dietary MBE and storage time was observed (*P* > 0.050). These results showed that dietary MBE (at 0.33 mg/kg) in finishing pigs would not influence the color change of pork throughout 6 d of refrigerated aerobic storage.

**Table 5:** Effect of dietary magnolia bark extract on the color stability and lipid oxidation of pork

Items	Dietary treatment (D)		Storage or incubation time (T)^1^			SEM	*P*-value		
	CON	MBE	0	1	2		D	T	D × T
Raw meat color descriptors									
L* (lightness)	55.0	52.0	51.0^b^	54.6^a^	54.8^a^	0.450	0.004	< 0.001	0.239
a* (redness)	5.85	5.65	7.71^a^	5.40^b^	4.14^c^	0.226	0.561	< 0.001	0.417
b* (yellowness)	6.41	5.87	7.75^a^	6.57^b^	4.10^c^	0.251	0.174	< 0.001	0.180
630/580 nm	1.24	1.25	1.35^a^	1.22^b^	1.16^c^	0.011	0.191	< 0.001	0.653
ΔE^2^	5.98	6.51	-	5.44	7.05	0.291	0.389	0.002	0.174
Lipid oxidation in meat, mg MDA/kg									
Raw	0.10	0.07	0.08^b^	0.09^b^	0.10^a^	0.004	0.039	< 0.001	0.407
Fe-Asc catalyzed	0.49	0.38	0.15^c^	0.39^b^	0.79^a^	0.041	0.049	< 0.001	0.034
Cooked	1.09	1.00	0.20^c^	1.09^b^	1.84^a^	0.091	0.189	< 0.001	0.260

*CON* Basal diet (control group), *Fe-Asc* Fe^3+^ and ascorbate oxidation catalyst, *MBE* Basal diet supplemented with 0.33 mg/kg of magnolia bark extract, *MDA* Malondialdehyde, *SEM* Standard error of the mean

^1^Times 0, 1, and 2 correspond to: 0, 3, and 6 d (raw meat); 0, 30, and 60 min (Fe-Asc catalyzed meat); 0, 2, and 4 d (cooked meat)

^2^Total color change between each day of storage and the day 0. Calculated as ΔE = [(ΔL*)^2^ + (Δa*)^2^ + (Δb*)^2^].^1/2^, where ΔL*, Δa* and Δb* are the differences in L*, a*, and b*, respectively, between day 0 and day 3 or 6

^a,b,c^Means with different superscript letter are significantly different within row

**Fig. 1:**
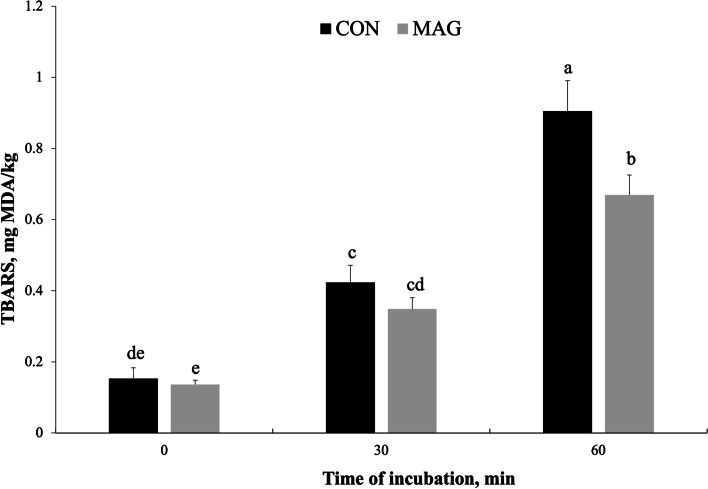
Interaction between dietary treatment and incubation time with Fe^3+^-ascorbate catalyst on pork lipid oxidation. CON: basal diet (control group); MBE: basal diet supplemented with 0.33 mg/kg of magnolia bark extract; MDA: malonaldehyde; TBARS: thiobarbituric acid reactive substances. ^a, b, c^^, d, e^Different letters indicate differences (*P* ≤ 0.05) between means. Columns are the mean values and error bars represent the standard error of the mean

The TBARS value is an overall measure of the secondary products of fatty acid peroxidation, associated to the development of rancidity off-flavors in meat [51]. In the present study, oxidative challenges were tested under different conditions (i.e., 6 d of aerobic storage, 60 min of Fe-Asc catalysis, and cooking plus 4 d of aerobic storage), each promoting lipid oxidation to a different extent. Testing meat oxidative stability under pro-oxidant challenges of varying nature and magnitude may offer some useful insights. For example, it has been demonstrated that diet-related differences in meat oxidative stability may remain masked under mild oxidative conditions (e.g., aerobic storage of raw meat), while becoming evident under more pro-oxidant challenges, such as incubation of meat homogenates with catalysts, or cooking followed by aerobic storage [52]. Indeed, in the present study, the lipid oxidation in raw pork increased quite gently after 6 d of storage (*P* < 0.001) but TBARS values never reached the rancidity perception threshold of 0.5 mg MDA/kg (reported by Sheard et al. [51]). Conversely, the extent of lipid oxidation constantly increased throughout 60 min of Fe-Asc catalysis and even a more pronounced development was observed over 4 d of storage after cooking (*P* < 0.001; Table 5). Likely, for raw pork, the amount of α-tocopherol measured in the present study ensured a good protection of lipids from oxidation, considering that 3 µg/g is close to the asymptotic limit of TBARS reduction in pork according to the prediction model of Sales and Koukolová [53]. Despite this, the raw meat of the MBE group had lower (*P* = 0.039) TBARS values than the control group (Table 5). The antioxidant effect of MBE supplementation was evident also in meat homogenates incubated with Fe-Asc in which an interaction between dietary treatment and incubation time was found (*P* = 0.034), with lower TBARS values being measured in the meat of MBE-fed pigs compared to the control group meat after 60 min of incubation, whereas no difference was observed after 0 and 30 min of incubation (Fig. 1).

Dietary MBE was thus able to reduce lipid oxidation in pork. Likewise, other studies reported a reduction of lipid oxidation in the fresh meat from pigs fed plant-origin supplements, such as *S. officinarum* extract [14], resveratrol (from *R. japonica*) [37], and quercetin (from *Styphnolobium japonicum* (L.) Schott) or *Origanum hirtum* Link essential oil [36]. Other studies also demonstrated that dietary supplementations with plant extracts, such as *Origanum vulgare* L. or *Melissa officinalis* L., reduced the TBARS production in Fe-Asc catalyzed pork homogenates [54]. The peculiar antioxidant properties of magnolol, honokiol, and the other bioactive molecules of MBE are well documented and form the basis of several potential therapeutical applications of magnolia bark, as concluded by both in vitro studies and in vivo studies on rodents [5]. In the light of the above discussed findings on the antioxidant capacity of meat, the antioxidant effect of MBE found in the present study might be linked to the effect of bioactive compounds of MBE on the endogenous enzymatic antioxidant defenses of muscle (increased CAT activity), which has been shown to reduce lipid oxidation in meat [55]. This hypothesis may be also supported by the observation that dietary MBE was ineffective in limiting TBARS production in cooked pork, with no difference (*P* > 0.050) between groups observed over 4 d of storage (Table 5). Cooking at 70 °C is likely to deactivate the enzymatic antioxidant systems, as recently demonstrated in chicken [56]. Similar to our results, Rossi et al. [12] observed that feeding pigs with *Lippia* spp. extract supplement reduced lipid oxidation of raw meat with no effect in meat cooked at 75 °C, although meat enzymatic activity was not investigated in their study.

## Conclusions

Supplementing the diet of finishing pigs with 0.33 mg/kg of magnolia bark extract reduced the lipid oxidation (assessed by TBARS analysis) in raw meat through aerobic refrigerated storage. This effect was probably due to the increased catalase activity observed in the meat from pigs fed MBE. No negative effects of dietary MBE on growth performance and carcass traits were observed. These results suggest that dietary MBE supplementation could have practical implications for a better shelf-life of pork. Further studies should investigate the effect of dietary MBE on meat protein oxidation, and test its supplementation at different doses.

## Data Availability

The datasets used and/or analyzed during the current study are available from the corresponding author on reasonable request.

## Abbreviations

630/580: Ratio between the reflectance spectrum at 630 nm and 580 nm
ΔE: Total color change
a*: Redness
ANOVA: Analysis of variance
b*: Yellowness
BHT: 3,5-Di-tert-4-butylhydroxytoluene
CAT: Catalase
DM: Dry matter
EDTA: Ethylenedinitrilotetraacetic acid
FA: Fatty acid
FRAP: Ferric reducing antioxidant power
Fe-Asc: Fe^3+^ and ascorbate oxidation catalyst
GPx: Glutathione peroxidase
h/H: Hypocholesterolemic to hypercholesterolemic ratio
i.d.: Internal diameter
L*: Lightness
LTL: *Longissimus thoracis et lumborum* Muscle
MBE: *Magnolia officinalis* (Rehder & E. Wilson) bark extract
MDA: Malondialdehyde
Nrf2: Nuclear factor E2-related factor 2
PTFE: Polytetrafluoroethylene
SOD: Superoxide dismutase
TBARS: Thiobarbituric acid reactive substances
TEAC: Trolox equivalent antioxidant capacity
U: Unit of enzymatic activity, µmol/min
UHPLC: Ultra-high performance liquid chromatography
